# Factors Predicting Ethiopian Anesthetists’ Intention to Leave Their Job

**DOI:** 10.1007/s00268-017-4318-7

**Published:** 2017-11-06

**Authors:** Adrienne Kols, Sharon Kibwana, Yohannes Molla, Firew Ayalew, Mihereteab Teshome, Jos van Roosmalen, Jelle Stekelenburg

**Affiliations:** 1 Jhpiego, 1615 Thames St # 200, Baltimore, MD 21231 USA; 2 Jhpiego Ethiopia, Kirkos Subcity, Kebele 02/03, House 693, Wollo Sefer, Addis Ababa, Ethiopia; 3 Athena Institute, VU University, Amsterdam, The Netherlands; 4 Department of Obstetrics and Gynecology, Leeuwarden Medical Centre, Leeuwarden, The Netherlands; 5 Department of Health Sciences, Global Health, University Medical Centre Groningen, University of Groningen, Groningen, The Netherlands

## Abstract

**Background:**

Ethiopia has rapidly expanded training programs for associate clinician anesthetists in order to address shortages of anesthesia providers. However, retaining them in the public health sector has proven challenging. This study aimed to determine anesthetists’ intentions to leave their jobs and identify factors that predict turnover intentions.

**Methods:**

A nationally representative, cross-sectional survey of 251 anesthetists working in public-sector hospitals in Ethiopia was conducted in 2014. Respondents were asked whether they planned to leave the job in the next year and what factors they considered important when making decisions to quit. Bivariate and multivariable logistic regressions were conducted to investigate 16 potential predictors of turnover intentions, including personal and facility characteristics as well as decision-making factors.

**Results:**

Almost half (*n* = 120; 47.8%) of anesthetists planned to leave their jobs in the next year, and turnover intentions peaked among those with 2–5 years of experience. Turnover intentions were not associated with the compulsory service obligation. Anesthetists rated salary and opportunities for professional development as the most important factors in decisions to quit. Five predictors of turnover intentions were significant in the multivariable model: younger age, working at a district rather than regional or referral hospital, the perceived importance of living conditions, opportunities for professional development, and conditions at the workplace.

**Conclusions:**

Human resources strategies focused on improving living conditions for anesthetists and expanding professional development opportunities may increase retention. Special attention should be focused on younger anesthetists and those posted at district hospitals.

**Electronic supplementary material:**

The online version of this article (doi:10.1007/s00268-017-4318-7) contains supplementary material, which is available to authorized users.

## Introduction

A shortage of anesthesia providers contributes to a crisis in access to surgical services in low- and middle-income countries (LMICs). The *Lancet* Commission on Global Surgery has estimated that 94% of the population in LMICs lacks access to safe, affordable anesthesia and surgical care [1]. Surgically treatable conditions, including in the field of obstetrics, however, make up an increasing proportion of the burden of disease in these settings [2]. To increase availability of anesthesia services, LMICs have shifted tasks from anesthesiologists to associate clinician cadres, such as anesthetists [3], and these providers now deliver most anesthesia services worldwide [4, 5]. But high turnover—driven by poor working conditions, limited remuneration, and low morale among other factors—has undermined efforts to expand the number of anesthesia providers in some LMICs [1, 6, 7].

Designing strategies to reduce turnover among anesthesia providers requires a good understanding of the local context because education, supervision, support, and working conditions vary widely [1, 4, 6–10]. Information about this essential cadre, however, remains limited [5]. This paper examines the situation in Ethiopia, where a shortage of anesthesia providers limits surgical and obstetric services [11]. Although estimates vary, there are certainly very few practicing physician anesthesiologists in Ethiopia and most hospitals rely exclusively on associate clinician anesthetists [11]. The Federal Ministry of Health (FMOH) has set explicit goals to increase the number of anesthetists by more than fivefold by 2025 [12]. To accomplish this, the FMOH expanded the number of pre-service education programs for anesthetists from two in 2006 to 26 in 2015 and introduced accelerated anesthetist training options for experienced nurses [13]. It has also implemented multiple interventions to strengthen the quality of anesthetist education.

Retaining newly trained anesthetists in the public sector has proven challenging [14, 15] despite a compulsory service obligation requiring anesthetists who graduate from public training institutions to serve 1–4 years at a public hospital, depending on the length of training, region, and sponsoring organization. This paper analyzes data on Ethiopian anesthetists’ turnover intentions, that is, whether they intend to leave their current job in the next year. This is widely accepted as the best available predictor of actual turnover [16]. We explore three questions: What proportion of anesthetists at public-sector hospitals in Ethiopia intend to leave their jobs in the next one year? What factors do they consider important when making decisions to remain in or leave their jobs? What factors predict anesthetists’ intentions to leave their job?

## Materials and methods

### Study design and sample

The Strengthening Human Resources for Health (HRH) Project (2012–2017) conducted a nationwide survey of job satisfaction, motivation, and turnover intentions among Ethiopian health workers at public-sector hospitals in 2014. A cross-sectional study design was used to gather nationally representative information on anesthetists at a randomly selected sample of 108 hospitals (out of the 122 total). Data collectors invited all eligible anesthetists at these hospitals to participate in the study. Anesthetists were eligible if they were full-time permanent hospital employees, had at least 6 months of work experience in anesthesia, and were available and willing to participate. Their training varied. Diploma Level 5 anesthetists are experienced nurses who have completed a 1-year Regional Health Science College training program. In the past, Advanced Diplomas were awarded to nurses with 2 years of anesthesia training, but this training track was discontinued. To receive a BSc in anesthesia, high school graduates must complete a 4-year university program, while experienced nurses must complete a 3-year university program. An MSc requires an additional 2-year university training program.

An adjusted minimum sample size of 232 anesthetists was calculated using statistical parameters of 95% confidence interval, expected proportion of intention to leave as 0.5, a design effect of 1.2, and a relative precision of 10%. On average, 2.4 anesthetists (range 1–10) were interviewed at each of 104 hospitals in 10 of Ethiopia’s 11 administrative regions and cities, for a total sample of 251 anesthetists; no anesthetists were available for interview at four hospitals.

### Data collection

Data were collected from May 28 to June 14, 2014. Individual interviews were conducted in private in the national language, Amharic. Interviewers read a series of closed-ended questions aloud to each respondent from a structured questionnaire and recorded their answers. The questionnaire was adapted from a job satisfaction and motivation tool developed by the Capacity Project for use with health workers in Uganda [17]. No systematic studies exist on the reliability and validity of the tool. However, health workers’ understanding of the questions was checked during a pretest of the tool in the Oromia Region, after which the wording and sequence of certain questions were revised to ensure their clarity and relevance to the Ethiopian setting.

Interviewers asked respondents about their demographic characteristics and work history, intentions to leave the job, and perceptions of conditions at home and at work that might affect retention. To assess turnover intentions, interviewers asked a yes-or-no question: “Are you planning to leave your job in the next one year?” To assess perceptions of 20 items that studies suggest may affect health workers’ decisions to leave their jobs, interviewers asked: “If you were planning to leave your job, how important would each of the following items be in that decision?” Respondents rated each item on a Likert scale, from 1 (not important) to 5 (extremely important).

All 24 interviewers had university degrees in health or social science, and one supervisor was assigned to each region. Supervisors were drawn from HRH Project staff. Data collectors and supervisors attended 2 days of training, which included role plays and practice interviews and covered consent procedures, research ethics, and interview techniques.

### Data analysis

Data were entered into Epi-Info and exported to STATA 13.1 for cleaning and analysis. Exploratory factor analysis and a literature review [18] were used to group 20 decision-making items into six uncorrelated factors: living conditions, conditions at the workplace, relationship with supervisor and co-workers, work burden, opportunities for professional development, and basic salary. To check reliability and internal consistency of items in each factor, we calculated Cronbach’s alpha coefficient [19]. Values exceeded the 0.7 cutoff for all factors except conditions at the workplace (*α* = 0.619). Chi-square test was used to test for associations between turnover intentions and demographic and work-related characteristics, with a *p* value <0.05 used to determine statistical significance.

Bivariate analysis assessed the independent effect of each predictor on turnover intentions. Then a multivariable logistic regression model was fitted, using a stepwise (backward) variable selection process to extract potential predictors of turnover intentions. After entering all variables into the model, stepwise selection was used to remove those where *p* > 0.3, leaving six variables. Multicollinearity among predictors was assessed to ensure reliability of the multivariable model. Adjusted odds ratios and 95% confidence intervals were calculated to show the magnitude of associations.

### Ethical considerations

The John Hopkins University Institutional Review Board approved the study protocol, and the Ethiopian FMOH gave permission to conduct the study. Before each interview, data collectors read a consent script in Amharic to obtain informed verbal consent. To ensure confidentiality, individual identifiers were not used during data collection and analysis.

## Results

### Respondents’ characteristics and turnover intentions

Most respondents had a bachelor’s degree (*n* = 145; 57.8%) (Table 1). They had served in the public health system from 6 months to 37 years (median = 4.8 years); for some, this included prior service as nurses. Almost two-thirds (*n* = 162; 64.5%) were currently fulfilling a compulsory service obligation, with 2–72 months remaining (data not shown).

**Table 1:** Distribution of respondents and percent who intend to leave their jobs in the next year, by socio-demographic and work-related characteristics

Characteristic	All respondents		Respondents who intend to leave their jobs in the next year		
	Number	Percent distribution (*n* = 251)	Number	% of total	*p* value*
Total	251	100	120	47.8	na
Gender					
Male	188	74.9	95	50.5	0.136
Female	63	25.1	25	39.7	
Age					
≤30 years	153	61.2	83	54.2	0.008
Over 30 years	97	38.8	36	37.1	
Marital status					
Single	135	53.8	70	51.8	0.055
Ever married	116	46.2	50	45.0	
Dependents					
Yes	191	76.1	83	43.5	0.014
No	60	23.9	37	61.7	
Educational qualification					
Master’s degree	18	7.2	5	27.8	0.104
Bachelor’s degree	145	57.8	71	49.0	
Advanced Diploma	36	14.3	14	38.9	
Diploma Level 5	52	20.7	30	57.7	
Years of service in public health system					
6 months–2 years	83	33.1	41	49.4	0.010
>2–5 years	53	21.1	34	64.2	
>5 years	115	45.8	45	39.1	
Currently fulfilling compulsory service obligation^a^					
Yes	162	64.5	78	48.1	0.575
No	89	35.5	42	47.2	
Type of facility					
District hospital	65	25.9	40	61.5	0.033
Regional/zonal hospital	94	37.4	42	44.7	
Referral hospital	92	36.6	38	41.3	

* Chi-square test

^a^The government requires anesthetists who graduate from a public educational institution to work (compulsory service) for 1–4 years in the public sector

Almost half (*n* = 120; 47.8%) planned to leave the job in the next year, but turnover intentions were significantly lower among those over age 30 (*n* = 36; 37.1%) and those with dependents (*n* = 83; 43.5%) (Table 1). Turnover intentions exceeded 60% among anesthetists with 2–5 years of service and those working at district hospitals. Fulfillment of the compulsory service obligation did not predict intention to leave.

### Factors affecting decisions to leave the job

Among the six decision-making factors, respondents rated basic salary (mean score = 4.52; SD = 1.0) and professional development opportunities (mean score = 4.22; SD = 0.89) as most important and living conditions as least important (mean score = 3.42, SD = 0.89). Conditions at the workplace (mean score = 3.72; SD = 0.94), work burden (mean score = 3.54; SD = 1.2), and relationship with supervisor and co-workers (mean score = 3.51; SD = 0.98) were of intermediate importance. Table 2 shows how respondents rated individual items. Over 85% said low pay, limited opportunities for promotion, poor access to higher education, safety concerns, and high cost of living were important.

**Table 2:** Percentage of anesthetists who consider items important in decisions to leave the job, mean and median scores

Items	*N*	% who say factor is important^a^	Score	
			Mean	Median
Living conditions				
High cost of living	251	87.2	4.07	5
Lack of housing facilities	246	82.9	3.82	4
Poor/lack of utilities (water, electricity) at home	249	78.7	3.49	4
Poor educational facilities for children^b^	163	77.9	3.58	4
Transportation problems	243	70.0	3.23	3
Work is far from home	218	58.3	2.93	3
Lack of access to telephones to stay in touch with family and friends	244	52.9	2.77	3
Conditions at the workplace				
Concerns about safety at work^c^	251	89.2	4.08	4
Poor access to supplies and equipment at work	251	79.7	3.63	4
Poor/lack of utilities (water, electricity, Internet) at work	251	74.9	3.45	4
Relationship with supervisor and co-workers				
Lack of recognition for good work	249	83.6	3.78	4
Unfair treatment by a supervisor	249	77.6	3.70	4
Poor supervision and feedback	249	74.3	3.29	3
Social conflicts in the workplace	249	73.9	3.28	4
Work burden				
Heavy workload	249	79.5	3.55	4
Long hours of work	251	78.5	3.54	4
Opportunities for professional development				
Poor access to higher education	250	93.6	4.44	5
Limited opportunities for promotion	250	92.4	4.32	5
Limited opportunities for in-service training	251	83.7	3.88	4
Basic salary				
Low pay	250	93.6	4.52	5

Response scale was: not important (1), somewhat important (2), important (3), very important (4), and extremely important (5)

^a^Includes responses of: extremely important, very important, and important

^b^Question was only asked of respondents with children

^c^Safety concerns focused on aging and unreliable anesthesia machines, exposure to infection due to lack of supplies, and physical trauma from moving heavy gas cylinders

The bivariate analysis found that turnover intentions were associated with two decision-making factors (living conditions and professional development opportunities) plus age, dependents, years of service, and facility type (Table 3). The final multivariable model included six variables, five of which were significant predictors of turnover intentions. Intention to leave the job fell by 5% with each additional year in age (adjusted OR = 0.95, 95% CI 0.92, 0.99). Anesthetists at referral hospitals were half as likely to intend to leave the job as those at district hospitals (adjusted OR = 0.48, 95% CI 0.23, 0.98). The more importance anesthetists attached to living conditions, the more likely they were to intend to quit (adjusted OR = 1.83, 95% CI 1.17, 2.86); the same was true for opportunities for professional development (adjusted OR = 1.91, 95% CI 1.26, 2.90). This pattern was reversed for conditions at the workplace: Anesthetists who considered this factor more important were *less* likely to intend to leave the job (adjusted OR = 0.63, 95% CI 0.4, 0.99).

**Table 3:** Bivariate and multivariable logistic regressions: intention to leave the job in the next year (outcome), by socio-demographic characteristics and importance of decision-making factors

Predictors	Bivariate logistic regression		Multivariable logistic regression^a^	
	Crude OR	95% CI	Adjusted OR	95% CI
Gender				
Male (ref.)	–			
Female	0.644	0.36–1.15		
Age in years	0.952	0.92–0.98	0.952	0.92–0.99
Marital status				
Single (ref.)				
Ever married	0.703	0.43–1.16		
Dependents				
No (ref.)				
Yes	0.478	0.26–0.87		
Educational qualification				
Master’s degree (ref.)				
Bachelor’s degree	2.494	0.85–7.36		
Diploma (Advanced or Level 5)	2.600	0.85–7.91		
Years of public health service	0.960	0.93–0.99		
Currently fulfilling compulsory service obligation				
No (ref.)				
Yes	1.039	0.62–1.74		
Type of facility				
District hospital (ref.)				
Regional/zonal hospital	0.505	0.27–0.96	0.525	0.26–1.05
Referral hospital	0.440	0.23–0.84	0.476	0.23–0.98
Importance of decision-making factors				
Living conditions	1.534	1.14–2.06	1.830	1.17–2.86
Conditions at workplace	1.195	0.92–1.56	0.627	0.40–0.99
Relationship with supervisor and co-workers	1.033	0.80–1.33		
Work burden	0.896	0.73–1.10	0.789	0.61–1.03
Professional development opportunities	1.752	1.27–2.42	1.912	1.26–2.90
Basic salary	1.213	0.94–1.57		

*OR* odds ratio, *CI* confidence interval

^a^All factors were entered into the multivariable model, and then stepwise selection was used to remove from the full model those with *p* > 0.3

## Discussion

Almost half (*n* = 120; 47.8%) of anesthetists said they planned to leave the job in the next year. Comparable data from other LMICs are rare, but a study in Thailand found similarly high levels of turnover intentions among nurse anesthetists [20]. Yet evidence suggests that most health workers do not act on stated turnover intentions. For example, actual annual turnover among midwives in Senegal was just 9%, even though 58.9% reported their intention to quit within a year [21]. In Ethiopia, 59.4% of all health workers at hospitals and health centers in Jimma Zone intended to leave their jobs in the next year or two compared with actual turnover of 45.9% over 5 years [22]. A record review in East Wollega Zone found an annual attrition rate of 19.3% for health workers holding diplomas or first degrees [23]. A 2012 baseline study conducted for the HRH Project found a 4% attrition rate for anesthetists in Ethiopia [24].

Even if personal relationships, limited job opportunities, or other factors prevent public-sector anesthetists from quitting, high turnover intentions signal that they are dissatisfied with their jobs and may be less motivated to perform well [25–27]. Therefore, the situation in Ethiopia demands action regardless of actual turnover levels, which this study did not document. The multivariable analysis identified five predictors of turnover intentions which can help devise effective strategies to reduce dissatisfaction and promote retention.

Intentions to leave the job declined with increasing age, confirming two studies of nurses in Ethiopia [14, 28]. Researchers have consistently found an inverse relationship between age and turnover intentions among nurses [18], and a multi-country study found that actual turnover among health workers over age 50 was half that of those under age 30 [25]. Younger health workers have fewer family ties and may leave a job to further their education or career. Compulsory service programs are designed to increase retention among this cohort, but have a mixed track record [29, 30]. In this study, the government service obligation did *not* predict turnover intentions, suggesting that other interventions are needed to retain younger anesthetists.

Turnover intentions were about twice as high at district hospitals as at regional and referral hospitals, even after controlling for variables related to living and working conditions. Attracting and retaining health workers at rural facilities like these is especially challenging and often requires special measures, such as recruiting students from and training them in the rural areas where they will be deployed, offering incentives for rural postings, and improving living and working conditions at rural facilities [31–33]. HR managers in Ethiopia should consider these kinds of interventions to retain anesthetists at district hospitals.

Turnover intentions increased with perceived importance of living conditions, which is consistent with previous studies. Inadequate living conditions increased job dissatisfaction and migration in Bangladesh [31], and housing had the greatest impact on non-physician health professionals choosing jobs at public health facilities in Mozambique [32]. The findings suggest that strategies such as housing allowances and improved access to water and electricity may reduce turnover intentions. Currently in Ethiopia, incentives related to housing are primarily directed to physicians.

Turnover intentions also increased with perceived importance of professional development opportunities. A study in Jimma Zone identified lack of ongoing skills development and training as leading reasons frustrating retention of hospital and health workers [22]. A survey of anesthetists at 41 hospitals in Ethiopia found they had very limited access to refresher training, continuing professional development, and support from a physician anesthesiologist [34]. Lack of coaching and continuing education are common concerns expressed by anesthesia providers in other LMICs as well [4, 35, 36]. The findings suggest that expanding opportunities for coaching, continuing education, and advanced degrees may promote anesthetists’ retention.

Contrary to expectations, turnover intentions declined with perceived importance of conditions at the workplace. Studies have documented serious deficits in supplies, equipment, and infrastructure for anesthesia in Ethiopia [11, 34], and the global literature suggests these deficits dampen morale of anesthetists in many LMICs [9, 35]. It is possible that the lack of internal consistency in the items making up this factor (which included safety and utilities as well as supplies and equipment) affected the results. Further investigation is needed to interpret this finding.

Researchers have applied the same tools and analytical approach used in this study to examine predictors of turnover intentions among nurses in Ethiopia [14]. Like anesthetists, half of nurses (*n* = 213; 50.2%) intended to leave the job in the next year. Few studies have compared turnover intentions of different health worker cadres, but they, too, found no significant differences [25, 37]. However, these studies only compared the levels, not the determinants, of turnover intentions. Notably, Ethiopian anesthetists have only one significant predictor of intention to leave in common with Ethiopian nurses: limited opportunities for professional development. For nurses, the two other significant predictors in a multivariable analysis were holding a university degree and fewer years of service. The divergence between anesthetists’ and nurses’ findings highlights the importance of separately assessing health worker cadres and tailoring retention strategies to each cadre’s different situation to ensure their effectiveness.

This study fills an important information gap, because anesthetists have been a largely overlooked and understudied cadre. The large sample provides nationally representative information for policy making, but has some limitations. It did not include the private sector or ask whether respondents intended to leave the facility, the public sector, the country, or the profession—which may be driven by different factors. A further round of data collection to determine whether anesthetists acted on their turnover intentions could have produced actionable information on individuals most at risk of quitting.

## Conclusions

High turnover intentions signal that anesthetists in Ethiopia are dissatisfied with their jobs and may suffer from low morale and motivation even if they do not actually quit. Findings suggest that the compulsory service requirement only delays anesthetists’ exit from the public health system and that salary is not a significant predictor of their decisions. Strategies that focus on improving living conditions for anesthetists and expanding opportunities for professional development are likely to prove more effective. Age and location also matter: Anesthetists are more likely to plan on quitting when they are young and if they are posted to district hospitals. HR managers should consider special measures for anesthetists in these vulnerable categories.

## Electronic supplementary material

Below is the link to the electronic supplementary material.

Supplementary material 1 (PDF 275 kb)
