# Supplementary material for: Factors Predicting Ethiopian Anesthetists’ Intention to Leave Their Job
Source: World J Surg. 2017 Nov 6;42(5):1262–9. doi: 10.1007/s00268-017-4318-7 (PMC5895675; doi:10.1007/s00268-017-4318-7)
Supplement: Supplementary file 1 — Supplementary material 1 (PDF 275 kb) [file 268_2017_4318_MOESM1_ESM.pdf]

ለጤና የሰው ኃይል የማጠናከር ፕሮጀክት

STRENGTHENING HUMAN RESOURCES FOR HEALTH PROJECT

በኢትዮጵያ ውስጥ በመንግስታዊ የጤና ዘርፍ የሚገኙ የጤና ባለሙያዎችን በቅጥር የማቆየት ሁኔታዎች ላይ ተጽዕኖ ያደርጋሉ ተብለው የሚታሰቡ ምክንያቶች፡- Perceptions of factors affecting the retention of public sector health workers in Ethiopia

ቅፅ 1፡ ለጤና ባለሙያዎች የተዘጋጀ መጠይቅ

Tool #1: Questionnaire for Health Workers

ለመሥሪያ ቤቱ አገልግሎት ብቻ

FOR OFFICIAL USE ONLY

የመጠይቁ ቁጥር:
Questionnaire Number ☐☐ ☐☐ ☐☐

የቃለ-መጠይቅ ቀን፡ ☐☐/☐☐/☐☐☐☐ (ቀን/ወር/ዓ.ም)
Date of Interview: (dd/mm/yyyy)

የጠያቂው ስም፡ ______________________________
Interviewer Name:

የተረጋገጠበት ቀን፡ ☐☐/☐☐/☐☐☐☐ (ቀን/ወር/ዓ.ም)
Date Checked: (dd/mm/yyyy)

የቡድን መሪ ስም፡ ______________________________
Team Leader Name:

ክፍል I: ጠቅላላ መረጃ

Section I: General information

ሀ) የክልል ስም፡ (Region name:) ____________________________

ለ) የዞን ስም፡ (Zone name:) ________________________________

ሐ) የወረዳ ስም፡ (Woreda name:) ___________________________

መ) የጤና ተቋሙ ዓይነት የጤና አመራር እርከን (አንዱን ያክብቡ)
(Type of facility or management structure) (circle one)

1= ዲስትሪክት ሆስፒታል (District hospital) 2=ዞናል/ሪጂናል ሆስፒታል (Dregional/ zonal hospital) 3= ሪፈራል ሆስፒታል (Referral hospital) 4= ጤና ጣቢያ (Health Center)

ሠ) ሙያዎት ምንድን ነው? (አንዱን ያክብቡ) (What is your profession?(Circle one))

1= ሐኪም (Medical Doctor)
2= ነርስ (Nurse)
3= አኔስቴቲስት (Anesthetist)
4= አዋላጅ (Midwife)
5= የጤና መኮንን (Health Officer)

ረ) የደረሱበት ከፍተኛ የትምህርት ደረጃ (አንዱን ያክብቡ)
(Highest level of your educational qualification) (Circle one)

1= የህክምና ዶክትሬት እና ስፔሺያላይዜሺን ሰርተፍኬት (MD with Specialty Certificate)
2= የህክምና ዶክትሬት ብቻ (MD only)
3= ማስትሬት (MA) ፣ የሳይንስ ማስትሬት (MSc) ፣የሕዝብ ጤና አጠባበቅ ማስትሬት (MPH), የጤና አጠባበቅ አስተዳደር ማስትሬት (MHA)
4= የሳይንስ ባችለር (BSc)
5= ከፍተኛ ዲፕሎማ (Advanced Diploma)
6= ዲፕሎማ /ደረጃ 4/ ደረጃ 5 (Diploma/LevelIV/LevelV)
7= ሌሎች (ይግለጹ) (Other Specify):___________________________

ሰ) አሁን ያሉበት የሙያ ስያሜ የሥራ ድርሻ ወይም መደብ (ከተያያዘው የኮድ ቁጥሩን ያስቀምጡ)፡
(Current job title) (enter code from the attached codebook:) ____________________________
____

ተ.ቁ(#)	ጥያቄዎች (*Question)*	መልሱን ይሙሉ ወይም ያክብቡ<br>*(Enter or circle answer)*	ወደ ሚቀጥለዉ ጥያቄ ዕለፍ<br>(Skip to next question)
1	የመልስ ሰጪውን/ዋን ፆታ ይግለፁ<br><br>(Observe gender of respondent and circle answer)	1= ወንድ (Male) 2= ሴት (Female)	
2	በየትኛው ዓ.ም ተወለዱ? (እንደ ኢትዮጵያዊያን አቆጣጠር)<br><br>(What is your birth year?) (in Ethiopian Calendar)	19☐☐ ዓ.ም(*yyyy*)	
3	የተወለዱበት ስፍራ የት ነው? (ገጠር ወይም ከተማ መሆኑን ያመልክቱ)<br><br>(What is your birth place?)<br>(Determine whether it is urban or rural and circle answer)	1= ከተማ (Urban)<br>2= ገጠር (Rural)	
3.1	የትኛው ክልል (Which region?)	______________________	
4	ስለትዳርዎ ሁኔታ ይግለፁ? (አንዱን ያክብቡ)<br><br>(What is your marital status?)*(Circle one)*	1= ያላገባ/ች (Single)<br>2= ያገባ/ች (Married)<br>3=የፈታ/ች (Divorced)<br>4=የሞተበት/ባት (Widowed)<br>5=የተለያየ/ች (Separated)	
5	ልጆች ወይንም በቤትዎ ወይም በውጭ የሚኖሩ በገንዘብ የሚረዷቸው ቤተሰቦች አሉዎት?<br><br>(Do you have children or dependents, living inside or outside your household, whom you financially support?)	1=አዎን (Yes) 2=የሉኝም (No)	መልሱ የሉኝም ከሆነ ወደ ጥያቄ 6 እለፍ<br><br>(If No, Skip to Q6)
5.1	ቁ.5ን «አዎን» ብለው ከመለሱ ስንት የሚረዷቸው ሰዎች አሉዎት?<br><br>**(If yes to Q5**: How many	☐☐	
5.2	ቁ.5ን «አዎን» ብለው ከመለሱ ከሁሉም በዕድሜው/ዋ ትንሽ የሆነው/ችው የሚረዱት/ዷት ስንት ዓመቱ/ቷ ነው? (ዕድሜ በዓመትና በወራት ይጥቀሱ)<br><br>**(If yes to Q5:** How old is the youngest dependent?)	☐☐ ዓመት ከ ☐☐ ወር<br>(*years*)<br>(*months*	
6	የጤና ባለሙያ ሁነዉ የተመረቁት ለመጀመሪያ ጊዜ ከየትኛዉ ተቋም ነው?<br><br>(From what institution did you receive your first professional qualification in health care?)		
7	የጤና ባለሙያ በመሆን የተመረቁት መቼ ነበር? (እንደ ኢትዮጵያውያን አቆጣጠር)<br><br>(When did you first qualify as a health professional?) (in Ethiopian Calendar)	☐☐☐☐ ዓ.ም ☐☐ ወር<br>*(yyyy)*<br>*months*	

7.1	ተጨማሪ የሙያ ትምህርት ወስደዉ ተመርቀዋል?<br>(Have you specialized or upgraded from your first professional qualification?)	1=አዎን (Yes) 2=አይደለም (No)	መልሱ አይደለም ከሆነ ወደ ጥያቄ 8 እለፍ<br>(If No, Skip to Q8)
7.2	የመጨረሻውን የጤና ባለሙያ ሁነዉ የተመረቁት መቼ ነበር? (እንደ ኢትዮጵያውያን አቆጣጠር)<br>(When did you receive your last qualification?) (in Ethiopian Calendar)?	☐☐☐☐ ዓ.ም (yyyy) ☐☐ ወር months	
8	የመጨረሻውን የሙያ ብቃት ስልጠና ካገኙ ወዲህ ይህ የመጀመሪያ ስራዎት ነው?<br>(Is this your first job since receiving your most recent qualification?)	1=አዎን (Yes) 2=አይደለም (No)	መልሱ አይደለም ከሆነ ወደ ጥያቄ 9 እለፍ
8.1	**ቁ.8ን «አይደለም»** ብለው ከመለሱ ከዚህ በፊት የነበሩበት ሥራ የት ነበር? የመንግስት ወይስ የግል ድርጅት ነበር?<br>**(If "No" to Q8:** Where was your last job? Was it in the public or private sector?)	1= የመንግስት (public) 2= የግል (private)	
8.2	**ቁ.8ን «አይደለም»** ብለው ከመለሱ ከዚህ በፊት የነበሩበት ሥራ የሚገኘው በገጠር ነበር ወይስ በከተማ?<br>**If "No" to Q8:** Was your last job located in an urban or rural area?	1= ከተማ (Urban) 2= ገጠር (Rural)	
8.3	**ቁ.8ን «አይደለም»** ብለው ከመለሱ ከዚህ በፊት የነበሩበት ሥራ ላይ ለምን ያህል ጊዜ አገለገሉ?<br>**If "No" to Q8:** How long did you serve in your last job?	☐☐ ዓመት years ከ ☐☐ ወር months	
8.4	**ቁ.8ን «አይደለም»** ብለው ከመለሱ ከዚህ በፊት የሚሰሩበትን ስራ ለመልቀቅ ዋነኛ ምክንያት ከነበሩት 3ቱን ይግለፁ<br>**(If "No" to Q8:**What were the three main reasons for leaving your last job?)	1.______________________________<br>2.______________________________<br>3. ______________________________	
9	አሁን ለሚያገለግሉበት የጤና ሙያ የሥራ/የሙያ ፈቃድ አለዎት?<br>(Do you have a license for your current professional qualification?)<br>ማስታወሻ: የሙያ ፈቃድ በ መልስ ሰጭዉ እጅ ባይኖር ችግር የለዉም<br>(Note: the credentials nay not be in the hand of the professional)	1=አዎን (Yes) 2=የለኝም (No)	መልሱ የሉኝም ከሆነነ ወደ ጥያቄ 10 እለፍ<br>(If No, Skip to Q10)

9.1	**ቁ.9ን «አዎን» ብለው ከመለሱ አሁን ለሚያገለግሉበት ሙያ (የጤና ባለሙያ ከሆኑ) መጨረሻ የሥራ ፈቃድ የተሰጥዎት መቼ ነበር? (እንደ ኢትዮጵያውያን አቆጣጠር)**<br><br>**(If yes to Q9**, when were you last licensed in your current profession (if you are a health professional)?	☐☐☐☐ ዓ.ም *(yyyy)*	
10	**በመንግስታዊ የጤና ዘርፍ ለምን ያህል ጊዜ አገልግለዋል?**<br><br>(How long have you worked for the public health system?)	☐☐ ዓመታት *years* ☐☐ ወራት *months*	
11	**በዚህ የጤና ተቋም ውስጥ ወይም የሥራ አመራር መዋቅር ላይ ለምን ያህል ጊዜ አገልግለዋል?**<br><br>(How long have you been working at this health facility or management structure?)	☐☐ ዓመታት *years* ☐☐ ወራት *months*	
12	**ምአሁኑ ወቅት በመንግስታዊ የጤና ዘርፍ ውስጥ እንዲያገለግሉ የሚያስገድዶዎት የአገልግሎት ግዳጅ አለ? (የገቡት የአገልግሎት ስምምነት)**<br><br>(Do you have a current obligation (compulsory service scheme) to work in the public health system?)<br><br>**<u>ማስታወሻ: ቀሪ አመታትን ብቻ ይመለከታል</u>**<br>(Note: Refer to the remaining years and /or months)	1=**አዎን** (Yes) 2=**የለም** (No)	**መልሱ የለም ከሆነነ ወደ ጥያቄ 13 እለፍ**<br><br>(If No, Skip to Q13)
12.1	**ቁ.12ን «አዎን» ብለው ከመለሱ ለስንት ዓመት የአገልግሎት ግዳጅ አለቦት?**<br><br>**(If"Yes" to Q12**: How many years of service are you obligated to perform?)	**ለ** ☐☐ **ዓመት** *years* **ከ** ☐☐ **ወር** *months*	
13	**አሁን የሚኖሩበት ቤት የባለቤትነት ይዞታ የማን ነው?**<br><br>What is the status of your current residential house? (Circle one)	1= **የግለ ወይም የራስ** (Own)<br>2= **ከመንግስት ኪራይ** (Rent from public)<br>3= **ከግለሰብ ኪራይ** (Rent from private)<br>4= **በጤና ተቋሙ የቀረበ (የተሰጠ)** (Provide by health facility)<br>5= **ከወላጆቼ (ከቤተሰብ) ጋር ነው የምኖረው** (Live with parents)	

ክፍል II: የልምድ መረጃ
Section II: Background Information

መልሶቹን በመጻፍ ወይም በማክበብ ያመልክቱ
Enter or circle the answers to the questions below.

ክፍል III: የስራ እርካታ
Section III: Job Satisfaction

አሁን የምጠይቅዎት ጥያቄ ስራዎትን በተመለከተ የሚሰማዎትን ስሜት ነው:: በእያንዳንዱ የሃሳብ መግለጫ ምን ያህል እንደሚስማሙና እንደማይስማሙ ከ1 እስከ 5 በሚለካ የመመዘኛ ነጥብ በመስጠት ይግለፁ፤

ይኸውም:-

5 = በጣም እስማማለሁ　4 = እስማማለሁ　3 = ገለልተኛ ነኝ

2 = አልስማማም　1 = ፈፅሞ አልስማማም

(Now I want to ask how you feel about your current job. Please tell me whether you agree or disagree with each statement, using a 5 point scale where:

5=strongly agree　4=agree　3=neutral　2=disagree　1=strongly disagree)

ተ.ቁ (#)	ከሚከተሉት የሃሳብ ነጥቦች ጋር ምን ያህል ይስማማሉ? **(To what extent do you agree or disagree with the following statements?)**	5= በጣም እስማማለሁ (Strongly agree)	4= እስማማለሁ (Agree)	3= ገለልተኛ ነኝ (Neutral)	2= አልስማማም (Disagree)	1= ፈፅሞ አልስማማም (Strongly disagree)
14	ሁሉን ነገር ሳገናዝብ፤ በስራዬ ደስተኛ ነኝ<br>(Considering everything, I am satisfied with my job.)	5	4	3	2	1
15	ደመወዜ በቂ (አግባብነት ያለው) ነው<br>(My salary package is fair.)	5	4	3	2	1
16	ተመሳሳይ የስራ ኃላፊነት ካላቸው ሌሎች ሠራተኞች ጋር ሳወዳድረው ደመወዜ ተመጣጣኝ ነው::<br>(My salary is fair compared to other staff with the same level of responsibility.)	5	4	3	2	1
17	እዚህ መስሪያ ቤት ውስጥ ዕድገት ለማግኘት በቂ ዕድል እንዳለኝ ይሰማኛል<br>(I feel there are sufficient opportunities for promotion with my employer.)	5	4	3	2	1
18	በኔ ደረጃ ካሉ ሌሎች ሠራተኞች ጋር ሲወዳደር የማገኘው ድጎማ (የትራንስፖርት፣ የሥራና የቤት አበል ወ.ዘ.ተ) ተመጣጣኝነት ያለው ነው::<br>(My benefits (such as transportation, duty allowance, housing, etc.) are fair compared with other staff at my level.)	5	4	3	2	1

ተ.ቁ (#)	ከሚከተሉት የሃሳብ ነጥቦች ጋር ምን ያህል ይስማማሉ?<br>**(To what extent do you agree or disagree with the following statements?)**	**5= በጣም እስማማለሁ (Strongly agree)**	**4= እስማማለሁ (Agree)**	**3= ገለልተኛ ነኝ (Neutral)**	**2= አልስማማም (Disagree)**	**1= ፈፅሞ አልስማማም (Strongly disagree)**
19	የምሰራው ስራ ከስራ ልምዴ ጋር እና ከክህሎቴ ጋር ተመጣጣኝነት ያለው ነው<br>(The job is a good match for my skills and experience).	5	4	3	2	1
20	የሥራ ድርሻዬ በግልጽ የተቀመጠና ወቅታዊ ነው::<br>(My job description is clear and up to date.)	5	4	3	2	1
21	ለምሰራው ጥሩ ስራ እውቅናን አገኛለሁ<br>(I receive recognition for doing good work.)	5	4	3	2	1
22	አለቃዬ የአስተዳደራዊ ፖሊሲዎችንና መመሪያዎችን በኔ ላይ ተፈፃሚ የሚያደርገው በአግባቡና ሚዛኑን በጠበቀ መልኩ ነው<br>(My supervisor applies personnel policies and practices fairly to me.)	5	4	3	2	1
23	ከአለቃዬ ጋር አብረን ያዘጋጀነው እና በስራ ላይ የምናውለው የስራ ዕቅድ አለኝ<br>(I have a current work plan developed with my supervisor.)	5	4	3	2	1
24	ዓመታዊ የስራ ክንውኔ የሚገመገመው በተሰጠኝ የስራ ዕቅድ መሠረት ነው<br>(My annual performance appraisal is based on my work plan.)	5	4	3	2	1
25	ድርጅቱ ለኔ ስራ ዋጋ እንደሚሰጥ ይሰማኛል::<br>(I feel that the organization values my work)	5	4	3	2	1
26	የሙያ ድጋፍ በሚያስፈልገኝ ጊዜ አለቃዬ ይተባበረኛል::<br>(My supervisor is available when I need support.)	5	4	3	2	1
27	ጓደኞቼን እና ቤተሰቤን እዚህ ቦታ ለሕክምና እንዲመጡ አበረታታቸዋለሁ::<br>(I would encourage my friends and family to seek care here.)	5	4	3	2	1
27.1	በምሰራበት ቦታ ስኬታማ ሊያረገኝ የሚችል ስልጠና ተሰጥቶኛል::<br>(I have been given the training that I need to succeed in my position.)	5	4	3	2	1
27.2	የሙያ ድጋፍ በሚያስፈልገኝ ጊዜ ምክር ማግኘት እችላለሁ::<br>(I have access to coaching and mentoring when needed.)	5	4	3	2	1

ተ.ቁ (#)	ከሚከተሉት የሃሳብ ነጥቦች ጋር ምን ያህል ይስማማሉ? **(To what extent do you agree or disagree with the following statements?)**	**5=** በጣም እስማማለሁ **(Strongly agree)**	**4=** እስማማለሁ **(Agree)**	**3=** ገለልተኛ ነኝ **(Neutral)**	**2=** አልስማማም **(Disagree)**	**1=** ፈፅሞ አልስማማም **(Strongly disagree)**
28	ተቋሙ ከኤች.አይ.ቪ/ኤድስና ሌሎች በስራ ላይ ከሚያጋጥሙ አደጋዎች እኔን ለመከላከል ተጨባጭ እርምጃዎችን ይወስዳል::<br>(The facility takes specific measures to protect me against HIV/AIDS and other occupational hazards)	5	4	3	2	1
29	እንደ ጤና ባለሙያ የማገለግለው ማህበረሰብ አካል እንደሆንኩ ይሰማኛል::<br>(I consider my self a part of the local community that I serve as a health worker.)	5	4	3	2	1
30	ማህበረሰቡ የእኔን አገልግሎት ዋጋ ይሰጠዋል ብዬ አምናለሁ::<br>(I feel that the community values my work.)	5	4	3	2	1
31	የዚህ ጤና ተቋም ኃላፊ ለስራው የተሰጠ እና የሙያ ብቃት ያለው ነው::<br>(The head of this health facility is competent and committed.)	5	4	3	2	1
31.1	ከሥራ ባልደረቦቼ ጋር ጥሩ ግንኙነት አለኝ::<br>I have a good relationship with co-workers.	5	4	3	2	1
32	በአጠቃላይ በምሰራበት ቡድን ወይም የስራ ባልደረቦቼ መካከል ያለው የሥራ ሞራል ጥሩ ነው::<br>(Overall, the morale level in my team or work group i s good.)	5	4	3	2	1
33	ቢያንስ ለሚቀጥሉት 2 ዓመታት እዚህ ቦታ ለመስራት አስባለሁ::<br>I intend to continue working here for at least 2 years.	5	4	3	2	1

ክፍል IV: የሥራና የኑሮ ሁኔታዎች

Section IV: Working and Living Conditions

አሁን የምጠይቅዎ በሚሠሩበት ተቋም ስለሚገኘው የሥራ ሁኔታ ነው:: በእያንዳንዱ ሃሳብ መግለጫ ምን ያህል እንደሚስማሙና እንደማይስማሙ ከ 1 እስከ 5 በሚለካ የመመዘኛ ነጥብ በመስጠት ይንገሩኝ:: ይኸውም:-

(Now I want to ask you about the working conditions at your current facility. Please tell me whether you agree or disagree with each statement, using a 5-point scale where:)

5= በጣም እስማማለሁ (strongly agree) 4=እስማማለሁ (agree) 3= ገለልተኛ ነኝ (neutral) 2= አልስማማም (disagree) 1= ፈፅሞ አልስማማም (strongly disagree)

ተ.ቁ (#)	ከሚከተሉት የመግለጫ ሃሳቦች ጋር ምን ያህል ይስማማሉ ወይም አይስማሙም?<br>**To what extent do you agree or disagree with the following statement?**	5= በጣም እስማማለሁ (Strongly agree)	4= እስማማለሁ (Agree)	3= ገለልተኛ ነኝ (Neutral)	2= አልስማማም (Disagree)	1= ፈፅሞ አልስማማም (Strongly disagree)	9=አይመለከተዉም (9=Note applicable)
34	ያለብኝ የስራ ጫና ልወጣው የምችለው ነው<br>(My work load is reasonable)	5	4	3	2	1	9
35	ስራዬን በአግባቡና ለአደጋ በማያጋልጥ መልኩ ለመወጣት የሚያስፈልጉኝ መሣሪያዎች (እንደ የእጅ ጓንት፣ መርፌዎች፣ ፋሻ፣ የቁስል መስፊያ ክር፣ ማጠቢያ መርዝ ወ.ዘ.ተ) በበቂ አቅርቦት አለኝ<br>(I have the supplies I need to do my job well and safely) (Such as gloves, needles, bandages, sutures, disinfectants).	5	4	3	2	1	9
36	ስራዬን በአግባቡና በቅልጥፍና ለመወጣት የምገለገልባቸው መሣሪያዎች (እንደ አልትራሳውንድ፣ ኤክስሬ ማሽን፣ የደም ግፊት መለኪያ ወ.ዘ.ተ) አቅርቦት አለኝ<br>(I have the working equipment I need to do my job well and efficiently) (Such as ultra sound, x-ray, blood pressure cuffs).	5	4	3	2	1	9
37	የምሰራበት ተቋም ጥሩ የመድኃኒቶች አቅርቦት አለው<br>(This facility has good access to drugs and medications.)	5	4	3	2	1	9
37.1	የምሰራበት ቦታ ንፁህ ነው<br>(My work space is clean)	5	4	3	2	1	9
38	አብዛኛውን ቀን ምሣዬን ለመብላት ጊዜ አለኝ<br>(I can take time to eat lunch almost everyday.)	5	4	3	2	1	9
39	በምኖርበት ቦታ ንፁህ የመጠጥ ውሃ አገኛለሁ<br>(At home, I have access to safe, clean water.)	5	4	3	2	1	9

ተ.ቁ (#)	ከሚከተሉት የመግለጫ ሃሳቦች ጋር ምን ያህል ይስማማሉ ወይም አይስማሙም?<br><br>**To what extent do you agree or disagree with the following statement?**	5= በጣም እስማማለሁ (Strongly agree)	4= እስማማለሁ (Agree)	3= ገለልተኛ ነኝ (Neutral)	2= አልስማማም (Disagree)	1= ፈፅሞ አልስማማም (Strongly disagree)	9=አይመለከተዉም (9=Note applicable)
40	በምስራበት ቦታ ንፁህ የመጠጥ ውሃ አገኛለሁ<br><br>(At work, I have access to safe, clean water.)	5	4	3	2	1	9
41	በምኖርበት ቦታ መብራት (የኤሌክትሪክ ኃይል) አለ<br><br>(At home, I have good access to electricity.)	5	4	3	2	1	9
42	በምስራበት ቦታ መብራት (የኤሌክትሪክ ኃይል) አለ<br><br>(At work, I have good access to electricity.)	5	4	3	2	1	9
43	በምስራበት ቦታ ፈጣን የኢንተርኔት አገልግሎት አለ<br><br>(At work, I have good internet connectivity)	5	4	3	2	1	9
44	ለልጆቼ ጥሩ ትምህርት ቤት አገኛለሁ<br><br>(I have access to good schooling for my children.)	5	4	3	2	1	9
45	ከቤት ወደ ስራ የምመላለስበት አስተማማኝና ቀልጣፋ የትራንስፖርት አገልግሎት አገኛለሁ<br><br>(I have safe and efficient transportation to work.)	5	4	3	2	1	9
46	ሥራዬን የማጣት /ከሥራ የመባረር ስጋት የለብኝም<br><br>(I am not worried about losing my job.)	5	4	3	2	1	9
47	የምስራበት ማህበረሰብ ጥሩ የሆነ የገበያ እና የመዝናኛ ማዕከል አለው<br>(The community where I live has good shopping and entertainment.)	5	4	3	2	1	9

ክፍል V: የክፍያና የድጎማዎች አስፈላጊነት

Section V: Importance of Compensation and Benefits

አሁን የምጠይቅዎ አሠሪዎች ለሠራተኞቻቸው የሚሰጧቸውን የክፍያና ድጎማ በተመለከተ ያለዎትን አስተያየት ነው። ከዚህ የሚከተሉት እያንዳንዱ እርስዎ በበኩልዎ እዚህ ስራ ላይ እንዲቆዩ የሚያደርጉዎትን ወሳኝነት አሏቸው የሚሏቸውን ነጥቦች ባለ 5 ነጥብ መመዘኛ በመጠቀም ይመልሱ።

ይኸውም፡-

5 = እጅግ በጣም አስፈላጊ (ወሳኝ) 4 = በጣም አስፈላጊ (ወሳኝ) 3 = አስፈላጊ (ወሳኝ)

2 = በመጠኑ አስፈላጊ 1 = አስፈላጊ ያልሆነ

(Next I want to ask your personal opinion about various compensation and benefits that employers may offer. How important is each of the following factors to you personally in deciding to stay in this job? Use a 5-point scale to answer, where:

5=extremely important, 4=very important, 3=important, 2=somewhat important, 1=not important)

ተ.ቁ (#)	እርስዎ በበኩልዎ የሚከተሉት ክፍያዎችና ድጎማዎች በስራ ላይ ለመቆየትዎ ምን ያህል ወሳኝነት አሏቸው? **(How important are the following compensation and benefits factors to you personally in deciding to stay in this job?**	5=እጅግ በጣም ወሳኝ አስፈላጊ (Extremely important)	4= በጣም ወሳኝ (very important)	3= አስፈላጊ ወሳኝ (impor tant)	2= በመጠኑ አስፈላገ somewhat important	1=አስፈላ ጊ ያልሆነ (Not important)
48	ደመወዝ (Salary)	5	4	3	2	1
49	ከሥራ ሲገለሉ የሚያገኟቸው ጥቅማ ጥቅሞች (እንደ ጡረታና፣ የአገልግሎት ክፍያ የመሳሰሉት) (Terminal benefits) (such as retirement and pension)	5	4	3	2	1
50	የቤት ኪራይ አበል ወይም ነፃ የመኖሪያ ቤት ማግኘ ት (Receiving a housing allowance or free housing)	5	4	3	2	1
51	የትራንስፖርት አበል ማግኘት (Assistance with transportation)	5	4	3	2	1
52	የአደጋ ተጋላጭነት አበል ማግኘት (Risk allowance)	5	4	3	2	1
53	የተረኛነት አበል ማግኘት (Duty allowance)	5	4	3	2	1
54	ለቤተሰብ ነፃ ህክምና ማግኘት (Health care for family)	5	4	3	2	1
55	ከሙያ የተነሳ የሚከሰት አደጋ ወይም ተጋላጭነት (Professional risk/hazard allowance)	5	4	3	2	1

56	**የምግብ አበል**<br>(Food allowance)<br>**ማስታወሻ፡ በተረኝነት ጊዜ በማዋለጃ፡ ኦፕሬሽን ክፍልና ወ.ዘ.ተ የምግብ አቅርቦት)**<br>(Note: Food allowance refers to catering service for staff on duty, maternity and OR, etc)	5	4	3	2	1

ክፍል VI: *ሥራን ለመልቀቅ የሚያበቁ ምክንያቶች*

Section VI: Factors that Affect the Decision to Leave a Job

በመቀጠል የምጠይቅዎት አሁን ባሉበት ሥራ ለመቆየት ወይም ያሉበትን ስራ ለመልቀቅ ሊያበቁ የሚችሉ ምክንያቶችን በተመለከተ ነው፡፡ አሁን ያሉበትን ሥራ ለመልቀቅ ቢያስቡ ከሚቀጥሉት ምክንያቶች የትኞቹ ለውሳኔዎት በጣም አስፈላጊ (አንገብጋቢ) የሚሆኑ ናቸው? ከ1 እስከ 5 በሚለካ የመመዘኛ ነጥብ ይግለፁ፡-

ይኸውም፡-
5=እጅግ በጣም አስፈላጊ (ወሳኝ) *4= በጣም አስፈላጊ ወሳኝ* *3= አስፈላጊ*
2=በመጠኑ አስፈላጊ *1=አስፈላጊ ያልሆነ*

(Now I want you to think about what factors might affect our decision to remain in your current job or to leave it. If you were to consider leaving your current job position, how important would each of the following factors be in that decision? Use a 3-point scale to answer, where:

5=extremely important *4=very important* *3=important*
2=somewhat important *1=not important*

ተ.ቁ (#)	ስራዎትን የመልቀቅ ሃሳብ ቢኖርዎ ከሚከተሉት ምክንያቶች የትኞቹ ለውሳኔዎት ምን ያህል ወሳኝነት ያላቸውና አስፈላጊ ናቸው?<br>**If you were to consider leaving your current job position, how important would the following factors be in that decision?**	**5=እጅግ በጣም አስፈላጊ (Extremely important)**	**4= በጣም አስፈላጊ (very important)**	**3= አስፈላገ (impor tant)**	**2= በመጠኑ አስፈላገ (Some what important)**	**1=አስፈላ ጊ ያልሆነ) (Not importa nt)**	**፱=አይመለከተዉም (፱=Not e applica ble)**
57	ዝቅተኛ ክፍያ (Low pay)	5	4	3	2	1	9
58	ከፍተኛ የስራ ጫና (መደራረብ) (Heavy workload)	5	4	3	2	1	9
58.1	ረዥም የሥራ ሰዓት (Long hours of work)	5	4	3	2	1	9
59	ከአለቃ የሚደርስ መድልዎ ፣በደል<br>(Unfair treatment by a supervisor)	5	4	3	2	1	9
60	በስራ ቦታ መሣሪያና የዕቃዎች አቅርቦት መጉደል (አለመሟላት)<br>(Poor access to supplies and equipment at work)	5	4	3	2	1	9
61	በስራ ላይ ለሚሆን ስልጠና በቂ ዕድል አለማግኘት<br>(Limited opportunities for in-service training)	5	4	3	2	1	9
62	የዕድገት በቂ ዕድል አለመኖር<br>(Limited opportunities for promotion)	5	4	3	2	1	9

ተ.ቁ (#)	ስራዎትን የመልቀቅ ሃሳብ ቢኖርዎ ከሚከተሉት ምክንያቶች የትኞቹ ለውሳኔዎት ምን ያህል ወሳኝነት ያላቸውና አስፈላጊ ናቸው?<br>**If you were to consider leaving your current job position, how important would the following factors be in that decision?**	**5=እጅግ በጣም አስፈላጊ (Extremely important)**	**4= በጣም አስፈላጊ (very important)**	**3= አስፈላገ (impor tant)**	**2= በመጠኑ አስፈላገ (Some what important)**	**1=አስፈላ ጊ ያልሆነ) (Not importa nt)**	**9=አይመለከ ተዉም (9=Not e applica ble)**
63	ለተከናወነ ጥሩ ሥራ እውቅና አለማግኘት<br>(Lack of recognition for good work done)	5	4	3	2	1	9
63.1	በሥራ ቦታ ላይ አለመግባባት<br>(Social conflicts in the workplace)	5	4	3	2	1	9
64	የቁጥጥር መላላትና ገንቢ የሥራ አስተያየት አለመኖር<br>(Poor supervision and feedback)	5	4	3	2	1	9
64.1	በስራ ላይ የደህንነት ዋስትና አለማግኘት (የአደጋ ስጋት)<br>(Concerns about safety at work)	5	4	3	2	1	9
65	የትራንስፖርት ችግር (Transportation problems)	5	4	3	2	1	9
66	በመኖሪያ ቤት የመሠረታዊ አገልግሎቶች (እንደ ውሃ፣ የኤሌክትሪክ ኃይል፣) አቅርቦት ችግር<br>(Poor/lack of utilities(water, electricity) at home)	5	4	3	2	1	9
67	በመስሪያ ቤት የመሠረታዊ አገልግሎቶች (ውሃ፣ ኤሌክትሪክ ኃይል፣ ኢንተርኔት) አቅርቦት ችግር<br>(Poor/lack of utilities (water, electricity, Internet) at work)	5	4	3	2	1	9
68	የመኖሪያ ቤት እጥረት (Lack of housing facilities)	5	4	3	2	1	9
69	ከቤተሰብና ወዳጆች ጋር በስልክ እጦት ምክንያት መገናኘት አለመቻል<br>(Access to telephones to stay in touch with family and friends)	5	4	3	2	1	9
70	የኑሮ ውድነት (High cost of living)	5	4	3	2	1	9
71	ለልጆች ተስማሚ ትምህርት ቤት አለመኖር<br>(Poor educational facilities for children)	5	4	3	2	1	9
72	ለራሴ የከፍተኛ ትምህርት ዕድል አለማግኘት<br>(Poor access to higher education for yourself)	5	4	3	2	1	9
73	የሥራ ቦታው ከቤቴ ርቀት አለው<br>(Work is far from home.)	5	4	3	2	1	9

ክፍል VII: የሥራ ቦታን የሚመለከቱ ተጨማሪ ጥያቄዎች

Section VII: Additional Questions about the Workplace

74. እዚህ ተቋም ውስጥ በስራ ላይ እንዲቆዩ ከሚያበረታቱ እና ደስ ከሚያሰኝዎት ነገሮች መካከል ሦስቱን ይግለፁ፡-

(Please identify three things that you like about working for this facility and that encourage you to continue working here:)

ሀ.____

ለ. ____

ሐ. ____

75. በዚህ መስሪያ ቤት ሲሰሩ ቅር የሚያሰኝዎትና ስራዎትን የመልቀቅ ሃሳብ ሊያነሳሱ ከሚችሉ ሦስቱን ነገሮችን ይጥቀሱ፡-

Please identify three things you don’t like about working here and that might make you consider leaving your job:

ሀ.____

ለ. ____

ሐ. ____

76. በሚቀጥለው አንድ ዓመት ውስጥ ስራዎን የመልቀቅ ሃሳብ አለዎት?

Are you planning to leave your job in the next one year?

1=አዎን (Yes) 2 =አይደለም (No)

77. **ቁ.76ን «አዎን»** ብለው ከመለሱ፤ ስራዎትን መልቀቅ እንዲያስቡ ያደረጉዎት ምክንያቶች ምንድን ናቸው?

If “Yes” to Q76: What are the main reasons you are planning to leave your job?

ሀ.____

ለ. ____

ሐ. ____

78. ስራዎትን ወይም የሚሰሩበትን ተቋም በተመለከተ በተጨማሪ መናገር የሚፈልጉት ነገር ይኖራል? እባክዎን ዘርዘር ያድርጉ።

Do you have anything else to say about your job or working conditions at this facility? Please be specific.

ሀ. ________________________________

ለ. ________________________________

ሐ. ________________________________

ጊዜ ስለሰጡንና ስለትብብርዎ እናመሰግናለን።

THANK YOU FOR YOUR TIME AND COOPERATION.
